# Supplementary material for: G-Protein-Coupled Estrogen Receptor (GPER)-Specific Agonist G1 Induces ER Stress Leading to Cell Death in MCF-7 Cells
Source: Biomolecules. 2019 Sep 18;9(9):503. doi: 10.3390/biom9090503 (PMC6769846; doi:10.3390/biom9090503)
Supplement: Supplementary file 1 [file biomolecules-09-00503-s001.pdf]

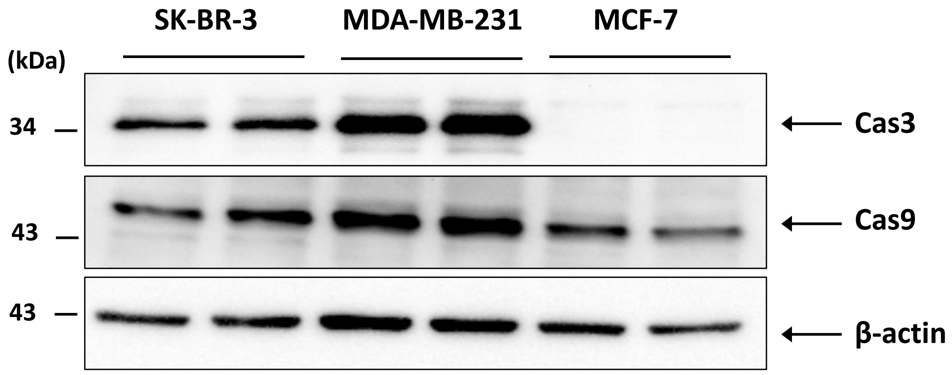

Figure S1. Caspase 3 was absent in MCF-7 cells. Total protein lysates from SK-BR-3, MDA-MB-231 and MCF-7 were subjected to Western blotting using appropriate antibodies as indicated.

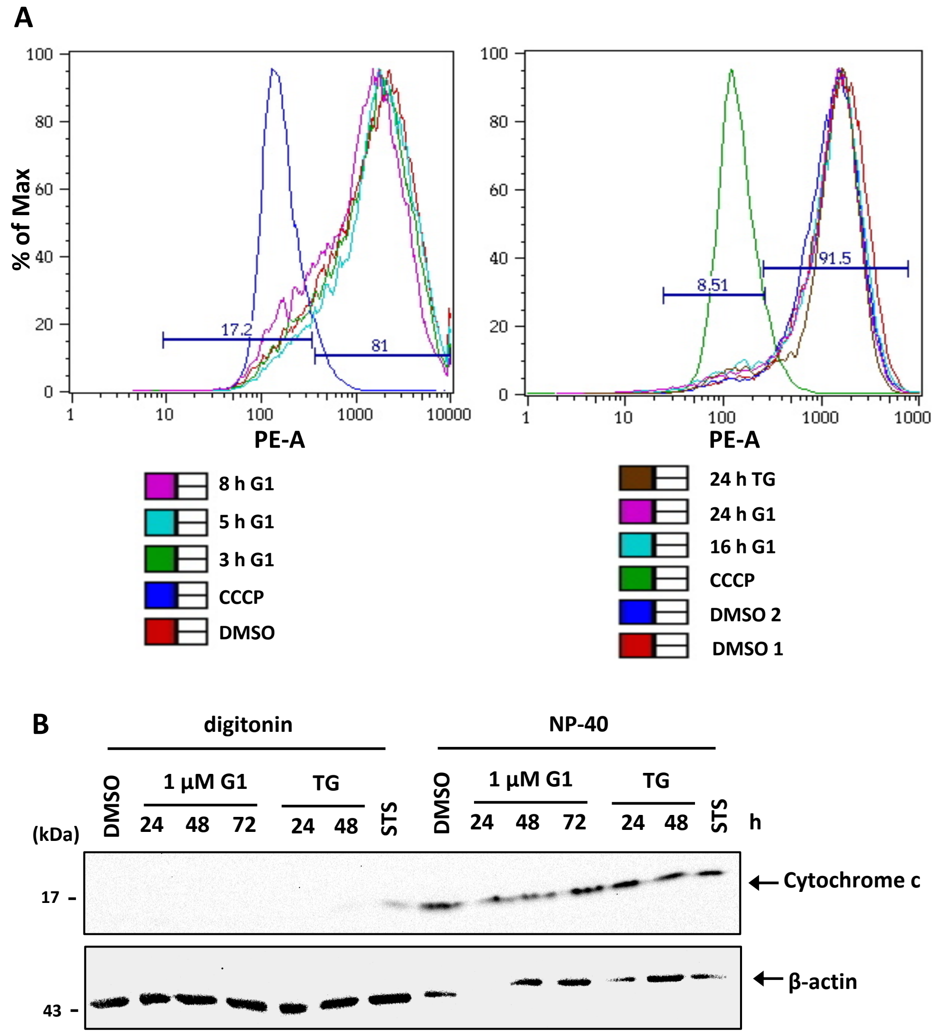

Figure S2. G1-induced cell death was not involved in the functional loss of mitochondrial potential. (A) MCF-7 cells were stimulated with 1 µM G1 or TG at different time points. Fluorescent signals of mitochondrial potential were measured by flow cytometry in TMRE-stained cells. CCCP was used as a positive control, which causes an uncoupling of mitochondria by inhibiting oxidative phosphorylation. (B) MCF-7 cells were treated with 1 µM G1 for 24, 48 and 72 h; 1 µM TG for 24 and 48 h or with 1 µM Staurosporine (STS) for 3 h. Subcellular fractionation was performed to collect cytosolic-enriched digitonin extract and membrane organellar protein-enriched NP-40 extract. Equal aliquots from each fraction were subjected to Western blotting using appropriate antibodies as indicated.
